# Evaluation of heat-treating heartworm-positive canine serum samples during treatment with Advantage Multi^®^ for Dogs and doxycycline

**DOI:** 10.1186/s13071-018-2685-z

**Published:** 2018-02-20

**Authors:** Molly D. Savadelis, Jennifer L. Roveto, Cameon M. Ohmes, Joe A. Hostetler, Terry L. Settje, Michael T. Dzimianski, Andrew R. Moorhead

**Affiliations:** 1 University of Georgia, College of Veterinary Medicine, Athens, GA USA; 2 Bayer Animal Health, Shawnee, KS USA

**Keywords:** Canine heartworm disease, *Dirofilaria immitis*, Heat-treatment, Macrocyclic lactone treatment, Moxidectin, Doxycycline, Advantage Multi®, Advocate®

## Abstract

**Background:**

The use of heat-treatment in canine and feline serum has been hypothesized to break the formation of antigen-antibody complexes, thereby freeing the heartworm antigen allowing for detection by commercially available heartworm antigen kits. While studies have analyzed the effect of heat-treating serum and plasma samples in the detection of heartworm antigen, these studies have not utilized necropsy verified results for validation. This study evaluated the use of heat-treating serum samples in experimentally infected dogs during adulticidal treatment in comparison with necropsy adult heartworm recovery.

**Methods:**

As part of a primary study, a total of 16 dogs were experimentally infected with 16 sexually mature adult heartworms using surgical transplantation, allocating 8 dogs in both the control and treated group. Treated dogs received 10 months of topical administration of Advantage Multi® for Dogs (10% Imidacloprid + 2.5% Moxidectin) every 4 weeks and 30 days of 10 mg/kg doxycycline BID. Blood samples were collected from all study animals prior to surgical transplantation of adult heartworms, on study days 0, 1, 3, 7, 14, 21, 28, and every 4 weeks thereafter for the duration of this study. Concentration of heartworm antigen was tested using the DiroCHEK® heartworm antigen test kit using serum samples both pre- and post-heat-treatment. Serum samples were heat-treated at 103 °C in a dry heat block for 10 min and centrifuging at 1818× *g* for 20 min.

**Results:**

There were a total of 4 instances (days 56, 140, 224 and 252) in 3 treated dogs in which a serum sample converted from negative for the detection of heartworm antigen prior to heat-treatment to positive for the detection of heartworm antigen post-heat-treatment. At necropsy, these dogs had no adult heartworms recovered and were all negative on antigen testing prior to and after heat treatment. There was 100% accuracy in the detection of either no infection, or 1–2 adult heartworm infections using the DiroCHEK in serum samples with and without heat-treatment at the time of necropsy.

**Conclusions:**

The DiroCHEK accurately diagnosed all dogs with live adults recovered at necropsy as heartworm antigen positive and all those dogs with no live adults recovered at necropsy as heartworm antigen negative without the use of heat-treatment for samples taken on the day of necropsy. Therefore, these results indicate that the use of heat-treating serum samples did not provide data of any additional value in the diagnosis of heartworm-positive dogs receiving treatment in this study. Additionally, these results may indicate that the conversion of serum samples from negative to positive for the presence of heartworm antigen with heat-treatment may not always accurately diagnose live adult heartworm infections since no adult heartworms were recovered at necropsy for those dogs in which a conversion event occurred. These conversion events may be detecting residual antigen leftover after all adult worms have died or may even be detecting off- target antigens, which have been denatured during heat-treatment. While a necropsy was not performed at the time of the conversion events, no live adult worms were recovered from any of the dogs in which a conversion event occurred earlier in treatment.

**Electronic supplementary material:**

The online version of this article (10.1186/s13071-018-2685-z) contains supplementary material, which is available to authorized users.

## Background

The use and efficacy of heat-treating serum and plasma samples for detecting heartworm antigen in both canines and felines have been debated. There is evidence that heat-treating serum samples may increase the level of detection of circulating heartworm antigen in canine and feline samples, therefore reducing the percentage of false negative test results [1]. The formation of inhibitory antigen-antibody complexes has been hypothesized to block the detection of free heartworm antigen, therefore resulting in false negative testing [2].

Dissociating antigen-antibody complex formations in accurate and sensitive antigen serological testing is not a new idea [3]. Many previous commercial heartworm testing procedures for heartworm included a dissociation step to destroy masking antibodies and inhibitory substances [4, 5]. This dissociation step was removed as commercially available heartworm antigen testing kits became more accurate and sensitive. Additionally, the interference of antigen-antibody complexes has been documented in serological testing for *Leishmania chagasi* infections causing visceral leishmaniasis. The use of acid dissociation in detecting *L. chagasi* antigen had a 3.5% conversion rate in which samples that had tested negative for the presence of *L. chagasi* tested positive for antigen after acid dissociation [6].

With the increasing use of various alternatives to the American Heartworm Society’s approved adulticidal treatment protocols for the treatment of canine heartworm disease, the utility and efficacy of heat-treating serum has been called into question [7]. Alternative canine heartworm adulticidal treatments, or slow-kill, utilize the long-term administration of macrocyclic lactones at prophylactic dosages with or without the use of doxycycline. Veterinarians rely on the accurate detection of circulating heartworm antigen to not only correctly diagnosis patients, but also to determine when a dog has been successfully cleared of adult heartworms. Drake et al. [8] evaluated 15 dogs previously treated using various slow-kill methods and found 53.3% of these dogs tested positive for the presence of heartworm antigen post-heat-treatment. Despite these antigen results, and due to the fact that these animals were client-owned, no necropsy was performed to confirm the presence or successful elimination of adult heartworms.

Recently, the efficacy of Advantage Multi® for Dogs (10% Imidacloprid + 2.5% Moxidectin) in combination with doxycycline has been evaluated using the aforementioned study animals [9]. The use of monthly topical administration of Advantage Multi® for Dogs for ten months along with 30 days of 10 mg/kg doxycycline BID was utilized in experimentally infected dogs. This study examines the use of heat-treating serum during heartworm treatment with corroborating necropsy results for comparison, allowing for a more in-depth analysis of differing heartworm antigen results and what status the test actually indicates. Moreover, in cases in which a heartworm antigen status converts from negative to positive with the use of heat-treatment, the question remains: is this due to the presence of live adult worms or residual antigen remaining after adult worm death and elimination?

## Methods

As part of a primary study, a total of 16 dogs with no previous exposure to any macrocyclic lactones were purchased from a supplier. The experimental groups consisted of non-treated controls and dogs treated with doxycycline and Advantage Multi® for Dogs (10% imidacloprid + 2.5% moxidectin) with 8 dogs in each group. Each study dog had a total of 16 adult heartworms surgically transplanted into the jugular vein, consisting of 11 females and 5 males [9, 10]. The treated dogs received 30 days of 10 mg/kg doxycycline BID and 10 monthly topical administrations of imidacloprid + moxidectin (IMD + MOX) every 4 weeks [9].

All study animals were tested for the presence of heartworm antigen utilizing the DiroCHEK® Heartworm Antigen Test Kit (Synbiotics Corporation, Zoetis, Kalamazoo, USA) according to manufacturer’s recommendation and then read on a spectrophotometer (Epoch, BioTek Instruments Inc., Winooski, VT, USA) at 490 nm. Each sample was analyzed pre- and post-heat-treatment. Serum samples were heat-treated at 103 °C in a dry heat-block for 10 min and then centrifuged at 1818× *g* for 20 min [1].

Serum samples for the detection of heartworm antigen were collected from each study animal prior to surgical transplant of adult heartworms (study day -35), post-surgical transplant (study day -9), at the initiation of treatment in the treated group (study day 0), study days 1, 3, 7, 14, 21, 28, and every four weeks thereafter throughout the remainder of the study.

All data analyses were performed using SAS v.9.3 software, utilizing an alpha level of 0.05 as significant. Optical density values of ≥ 0.0652 were identified as heartworm-positive and optical density values of ≤ 0.052 were identified as heartworm-negative. These optical density cut-off values were calculated by assessing non-infected and naturally-infected heartworm-positive canine serum samples on the DiroCHEK®. A total of 52 heartworm-positive samples and 74 heartworm-negative samples were used to calculate the average optical density reading and standard deviation for each group. Samples were initially analyzed for visual color change as intended by the manufacturer and then read using a spectrophotometer at 490 nm.

## Results

**Fig. 1:**
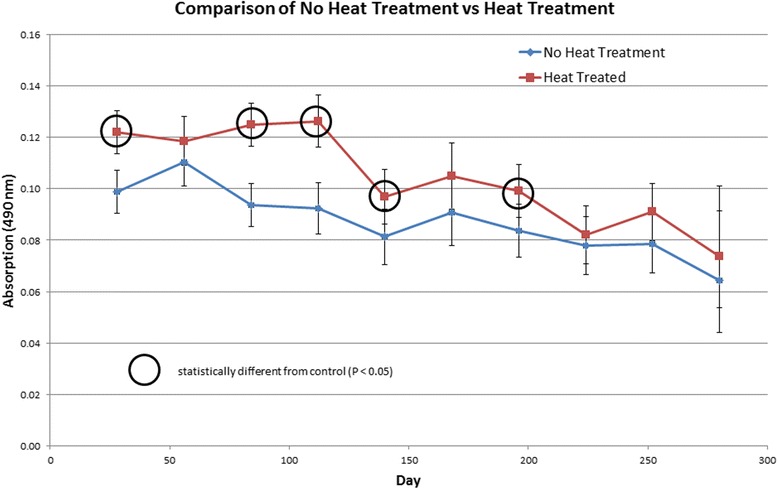
Serum samples were collected from all study dogs monthly throughout this study. Heartworm antigen concentration was tested using the DiroCHEK® for both pre- and post-heat-treatment of samples. The optical density for both samples was read at 490 nm. No significant differences were found between these two methods with respect to the distribution of categorical labeled negative, slightly positive and positive antigen results according to visual color change. However, analyses of the actual optical density values resulted in absorption values post-heat-treatment as being significantly higher (*P* < 0.05) than those results pre-heat-treatment on study days 28 (*t*_(29)_ = 3.98, *P* = 0.0004), 84 (*t*_(30)_ = 5.40, *P* = <0.0001), 112 (*t*_(30)_ = 4.89, *P* = <0.0001), 140 (*t*_(32)_ = 2.09, *P* = 0.0450) and 196 (*t*_(34)_ = 2.17, *P* = 0.0371) using a repeated measures analysis of variance having a significant treatment by time interaction (degrees of freedom were rounded for display purposes). Overall, the optical density readings for heat-treated samples were higher than non-heat-treated samples

Prior to the surgical transplant of adult heartworms on study day -35, no heartworm antigen was detected in any of the study dogs. By study day 0, with the initiation of doxycycline and IMD + MOX in the treated group, all dogs tested positive for the presence of heartworm antigen. Post-heat-treatment, serum samples resulted in higher optical density values as compared to serum samples not heat-treated. Heat-treated samples had significantly higher optical density values than non-heat-treated samples on study days 28 (*t*_(29)_ = 3.98, *P* = 0.0004), 84 (*t*_(30)_ = 5.40, *P* = <0.0001), 112 (*t*_(30)_ = 4.89, *P* = <0.0001), 140 (*t*_(32)_ = 2.09, *P* = 0.0450) and 196 (*t*_(34)_ = 2.17, *P* = 0.0371) using a repeated measures analysis of variance (Fig. 1).

**Fig. 2:**
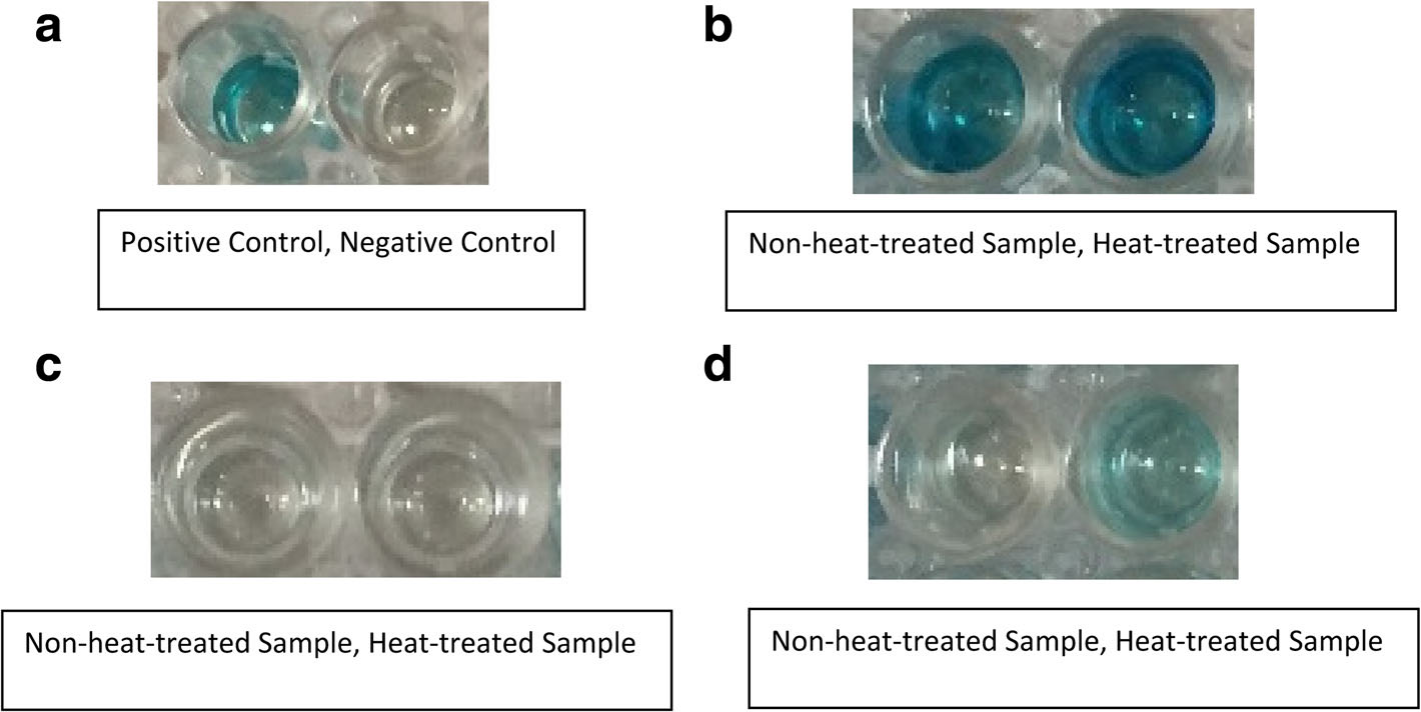
DiroCHEK heartworm antigen result plate. Blue color indicates the presence of heartworm antigen. The intensity of color change corresponds to the concentration of heartworm antigen present. **a** Each kit comes with a positive and negative control sample dropper to be run with each sample assay. **b** There is no visual difference between the pre-heat-treatment sample and the post-heat-treatment sample for this dog. **c** In truly heartworm negative samples, heat-treatment does not cause any color change. **d** This sample converted from negative to positive post-heat-treatment as indicated by the color change

A total of 4 samples from 3 different dogs had instances in which the sample converted from testing negative for the presence of heartworm antigen without heat-treatment to testing positive for the presence of heartworm antigen with heat-treatment by visual interpretation of test results. These negative to positive conversion events are defined as the presence or absence of a color-change in the test well using the DiroCHEK® (Fig. 2). All conversion events occurred in the treated group on study days 56, 140, 224 and 252, with the same dog exhibiting a conversion event on study days 56 and 224.

**Fig. 3:**
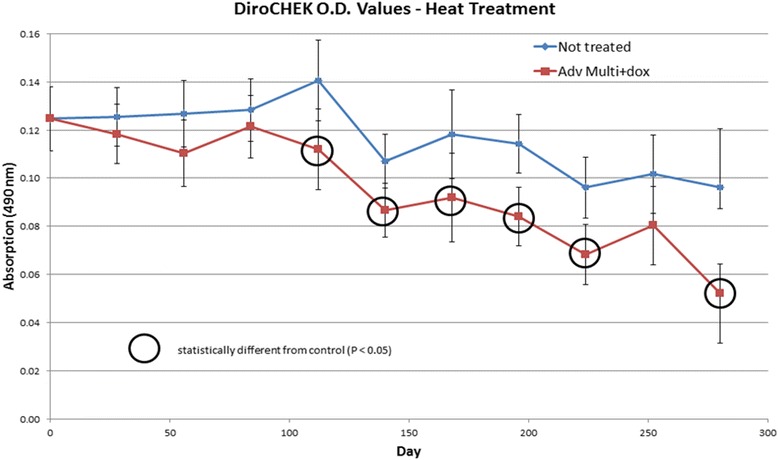
Serum samples were tested for the presence of heartworm antigen monthly pre- and post-heat-treatment. Heat-treated samples were placed at 103 °C for 10 min and centrifuged at 1818× *g* for 20 min. The absorption values using heat-treatment were significantly lower (*P* < 0.05) from animals in the treated group as compared to the non-treated animals on study days 112 (*t*_(17)_ = 2.56, *P* = 0.0205), 140 (*t*_(22)_ = 2.69, *P* = 0.0133), 168 (*t*_(18)_ = 2.12, *P* = 0.0479), 196 (*t*_(23)_ = 3.63, *P* = 0.0014), 224 (*t*_(20)_ = 3.26, *P* = 0.0039), 279 (*t*_(4)_ = 6.76, *P* = 0.0031), 280 (*t*_(4)_ = 5.16, *P* = 0.0074) and 281 (*t*_(4)_ = 6.87, *P* = 0.0016) using a repeated measures analysis of variance having a significant treatment by time interaction (degrees of freedom were rounded for display purposes)

**Fig. 4:**
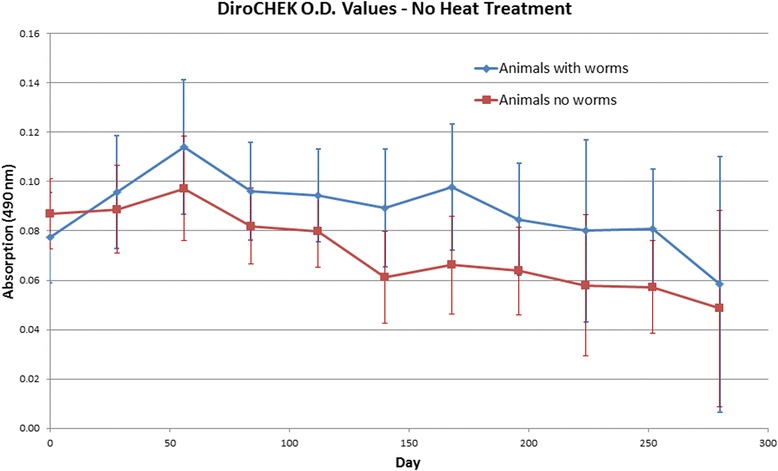
Serum samples were tested for the presence of heartworm antigen monthly pre- and post-heat-treatment. The optical density for both samples was read at 490 nm. The optical density reading for the 8 dogs in the IMD + MOX and doxycycline treated group differed between those dogs in which adult heartworms were recovered at necropsy and those dogs in which no adult heartworms were recovered. For dogs in the treated group in which adult heartworms were recovered, the optical density using the DiroCHEK tested higher than those treated dogs in which no adult heartworms were recovered

Optical density values in the serum samples collected from the IMD + MOX and doxycycline treated group generally began to decline after study day 86, 3 months post-treatment, both pre- and post-heat-treatment (Fig. 3) [9]. Non-treated control dogs remained positive for the presence of heartworm antigen throughout the study, while 5 of the 8 IMD + MOX and doxycycline treated group dogs tested negative for the presence of heartworm antigen and confirmed at necropsy at the termination of the study on Study Days 279–282 (10 months post-treatment). Comparing necropsy adult heartworm recovery to end-of-study heartworm antigen results, all heartworm antigen test results correlated with the necropsy adult heartworm recovery for each study dog (Additional file 1: Figures S1-S4). All 5 IMD + MOX and doxycycline-treated dogs testing negative for the presence of adult heartworms did not have any adults recovered at necropsy, while the remaining 3 IMD + MOX and doxycycline treated dogs that remained positive for the presence of heartworm antigen had 1–2 adult heartworms recovered at necropsy [9] (Table 1, Fig. 4). The 3 IMD + MOX and doxycycline treated dogs in which a conversion event post heat-treatment occurred during the course of treatment, had no adult heartworms recovered at necropsy.

**Table 1:** Adult heartworm status as compared to necropsy adult heartworm recovery. Serum samples were collected immediately prior to euthanasia on study days 278–282 (10 months post-treatment). Heartworm antigen concentration was tested utilizing the DiroCHEK® heartworm antigen test kit. Heartworm antigen status was determined according to color change as recommended by the manufacturer’s recommendations. All heartworm antigen testing performed immediately prior to euthanasia accurately diagnosed live heartworm infection status. Heartworm antigen status recorded represents both pre- and post-heat-treated antigen results

Group	Dog ID	Heartworm antigen status	Adult heartworm recovery
Control	43493	Positive	10
	43491	Positive	10
	43486	Positive	11
	43481	Positive	11
	43492	Positive	10
	43480	Positive	12
	43489	Positive	10
	43484	Positive	11
IMD + MOX and doxycycline	43483	Positive	2
	43485	Positive	1
	43495	Positive	2
	43494	Negative	0
	43382^a^	Negative	0
	43490^a^	Negative	0
	43488	Negative	0
	43487^a^	Negative	0

^a^Study animal’s serum sample converted from heartworm negative to positive post-heat-treatment at some point during this study treatment timeline

## Discussion

All serum sample data generated by the DiroCHEK® heartworm antigen test kit was recorded visually as the manufacturers’ recommendations specify, with the addition of using a spectrophotometer reading for optical density. Visual interpretation of the DiroCHEK coincided with the optical density reading results. Outside of the four conversion events in which a sample converted from negative to positive post-heat-treatment, the color change intensity very rarely differed between pre- and post-heat-treatment samples (Fig. 2). Therefore in a clinic setting, without a spectrophotometer, there will likely be no visual difference between pre- and post-heat-treatment heartworm antigen concentration that can be detected outside of antigen conversion events.

While a necropsy was not performed at the time in which a conversion event occurred post-heat-treatment, all dogs that tested negative for the presence of adult heartworm antigen pre- and post-heat-treatment did not have any adult heartworms present during necropsy. Despite post-heat-treatment serum samples occasionally taking longer to test negative for the presence of heartworm antigen as compared to pre-heat-treatment samples, both samples accurately diagnosed the successful elimination of adult heartworms. At the time of necropsy, all serum samples that tested negative for the presence of heartworm antigen pre- and post-heat-treatment did not have any live adult worms recovered. In time, all three dogs in which an antigen conversion event occurred, tested negative for the presence of heartworm antigen. This may indicate that heartworm antigen conversion events may not be accurately diagnosing the presence of live adult heartworms at the time of sampling but instead be detecting low levels of residual antigen after adult heartworm death.

It is unknown exactly how long antigen remains circulating in the blood stream after the death of adult heartworms. Previous studies evaluating the efficacy of melarsomine dihydrochloride in field studies found that 98.2% of all dogs treated with either 2.2 mg/kg or 2.5 mg/kg melarsomine dihydrochloride tested negative for the presence of heartworm antigen 90 days after adulticide treatment using the Pet-Check (IDEXX, Westbrook, ME, USA). These samples did not have a dissociation step to break antigen-antibody complexes. While it is possible that the concentration of circulating adult heartworm antibody increases during treatment, potentially blocking the detection of free adult heartworm antigen by commercially available antigen test kits, heat-treatment may allow detection of residual adult heartworm antigen post-adult heartworm elimination with either melarsomine or slow-kill.

In the IMD + MOX and doxycycline treated group, the three dogs in which adult heartworms were recovered during necropsy tested positive for the presence of adult heartworm antigen both pre- and post-heat-treatment of samples. At no point during the treatment did these three dog samples test negative for the presence of adult heartworm antigens pre- or post-heat-treatment. All IMD + MOX and doxycycline treated dogs in which no adult heartworms were recovered at necropsy, tested negative for the presence of adult heartworm antigens both pre- and post-heat-treatment at varying times prior to necropsy, but all dogs tested negative the day of necropsy both pre- and post-heat-treatment. Therefore, all heat-treated heartworm negative samples were truly indicative of no live adult heartworms present. These results indicate that the use of heat-treated serum samples for the detection of heartworm antigen did not provide additional post-treatment information, of clinical value, in the diagnosis of live adult heartworms.

Recent studies have evaluated the potential for antigen cross-reactivity with various commercially available heartworm antigen test kits pre- and post-heat-treatment of samples. In one such study, live adults of *Dirofilaria immitis*, *D. repens*, *Toxocara canis*, *T. cati*, *Dipylidium caninum*, *Taenia taeniformis* and *Mesocestoides* sp. larvae were incubated in saline for 30 min. The saline solutions were then evaluated using the SNAP® HTWM (IDEXX), SNAP® 4Dx® (IDEXX, Westbrook, Maine, USA), WITNESS® HW (IDEXX, Westbrook, Maine, USA), Speed Diro™ (Virbac, Fort Worth, Texas, USA), PetChek® (IDEXX, Westbrook, Maine, USA), and the DiroCHEK® (Zoetis, Kalamazoo, USA). Cross reactivity of these saline solutions was found with *D. repens*, *Toxocara canis*, *T. cati*, *D. canium* and *Taenia taeniformis* [11]. Additionally, serum from dogs naturally infected with *A. vasorum* or *D. repens* living in heartworm transmission-free areas were tested pre- and post-heat-treatment using the same commercially available heartworm tests as mentioned before. Dogs infected with *D. repens* tested positive for cross-reactivity pre-heat-treatment using the WITNESS® HW and DiroCHEK®. Of these same samples, all tested positive for cross reactivity post-heat-treatment. Dogs infected with *A. vasorum* tested positive for cross reactivity pre-heat-treatment using the SNAP® HTWM, PetChek®, and a few on WITNESS® and DiroCHEK. Of these dogs, an increased number of samples tested positive for cross reactivity post-heat-treatment for all the heartworm antigen tests [11]. These data indicate that the potential for cross-reactivity in false positive heartworm antigen testing does play a role and that the heat-treatment of samples increases the rate of false positive heartworm test results by decreasing specificity.

## Conclusions

This study provides compelling evidence that while the use of heat-treatment may allow increased detection of circulating heartworm antigen and diagnosis of heartworm infection, we still do not fully understand the effects and mechanism of heat-treating samples. We hypothesize heat-treatment may release residual antigen after adult worm death and elimination or may even detect off target epitopes due to protein denaturing at 103 °C that can cross-react. The 100% accuracy of the DiroCHEK® Heartworm Antigen Test Kit to detect the presence of live adult heartworms without the need for heat-treatment indicates that the use of heat-treatment in this study did not provide any unique or valuable data in the detection of viable heartworm infection post-treatment.

## Additional files

Additional file 1: Figure S1. DiroCHEK Heartworm Antigen test results for dogs 43491, 43493, 43494 and 43495 with adult heartworm recoveries of 10, 10, 0 and 2 adults, respectively. The top two wells contain the positive and negative controls. There are two wells per dog with the first well containing the pre-heat-treatment serum sample and the second well containing the post-heat-treatment serum sample for each dog. **Figure S2.** DiroCHEK Heartworm Antigen test results for dogs 43481, 43482, 43485 and 43486 with adult heartworm recoveries of 11, 0, 1 and 11 adults, respectively. The top two wells contain the positive and negative controls. There are two wells per dog with the first well containing the pre-heat-treatment serum sample and the second well containing the post-heat-treatment serum sample for each dog. **Figure S3.** DiroCHEK Heartworm Antigen test results for dogs 43480, 43483, 43490 and 43492 with adult heartworm recoveries of 12, 2, 0 and 10 adults, respectively. The top two wells contain the positive and negative controls. There are two wells per dog with the first well containing the pre-heat-treatment serum sample and the second well containing the post-heat-treatment serum sample for each dog. **Figure S4.** DiroCHEK Heartworm Antigen test results for dogs 43484, 43487, 43488 and 43489 with adult heartworm recoveries of 11, 0, 0 and 10 adults, respectively. The top two wells contain the positive and negative controls. There are two wells per dog with the first well containing the pre-heat-treatment serum sample and the second well containing the post-heat-treatment serum sample for each dog. (ZIP 39142 kb)

## Abbreviation

IMD + MOX: Advantage Multi® for Dogs (10% imidacloprid + 2.5% moxidectin) topical solution
